# Supplementary material for: Glucose Starvation-Induced Rapid Death of Nrf1α-Deficient, but Not Nrf2-Deficient, Hepatoma Cells Results from Its Fatal Defects in the Redox Metabolism Reprogramming
Source: Oxid Med Cell Longev. 2020 Jul 28;2020:4959821. doi: 10.1155/2020/4959821 (PMC7407023; doi:10.1155/2020/4959821)
Supplement: Supplementary materials — Figure S1: distinct cellular responses to glucose deprivation. Figure S2: the quantified results of western blot data in Figures 3, 4, and 5 by Quantity One 4.5.2 software. Figure S3: time-dependent effects of glucose deprivation on Nrf1 and Nrf2 with distinct half-lives. Figure S4: the mRNA expression of CYBA, NOXO1, and RAC1 in WT, Nrf1a-/-, Nrf1a-/- + siNrf2, Nrf2-/-, and caNrf2 from transcriptomic sequencing. Table S1: the key resources used in this work. [file 4959821.f1.pdf]

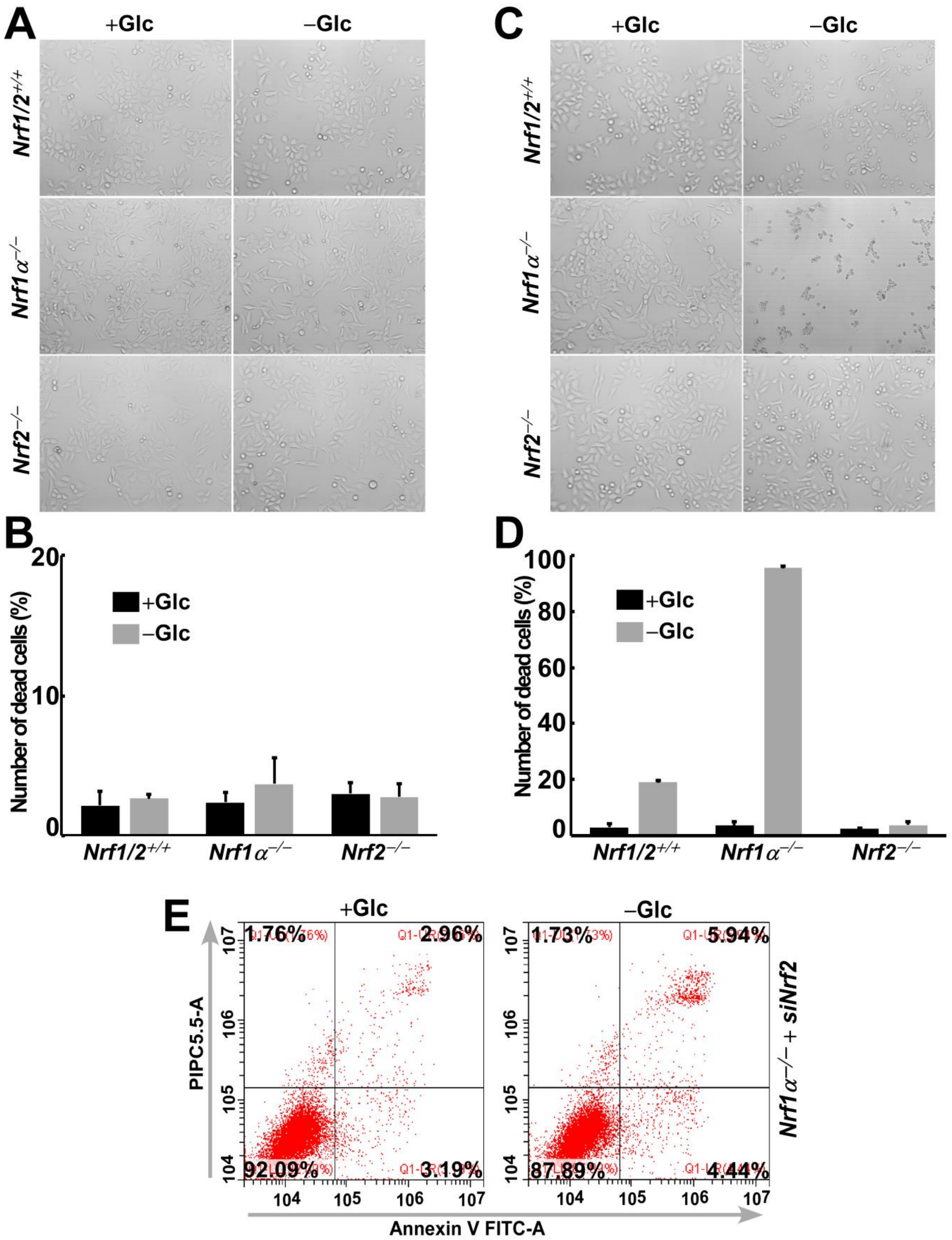

Figure S1. Distinct cellular responses to glucose deprivation.

(A,B) *Nrf1/2*$^{+/+}$, *Nrf1α*$^{-/-}$ and *Nrf2*$^{-/-}$ cells had been starved, or not starved, in glucose-free media for 6 h, before their morphological observation by microscopy (*A*, with an original magnification of 200×) and counting of dead cells by trypan blue staining (*B*).

(C,D) The above experimental cells were or were not subjected to glucose starvation for 24 h, before morphological observation (*C*) and dead cell counting (*D*).

(E) Apoptosis of *Nrf1α*$^{-/-}$ + *siNrf2* cells, that had been glucose-starved for 12 h, were determined by Flow cytometry.

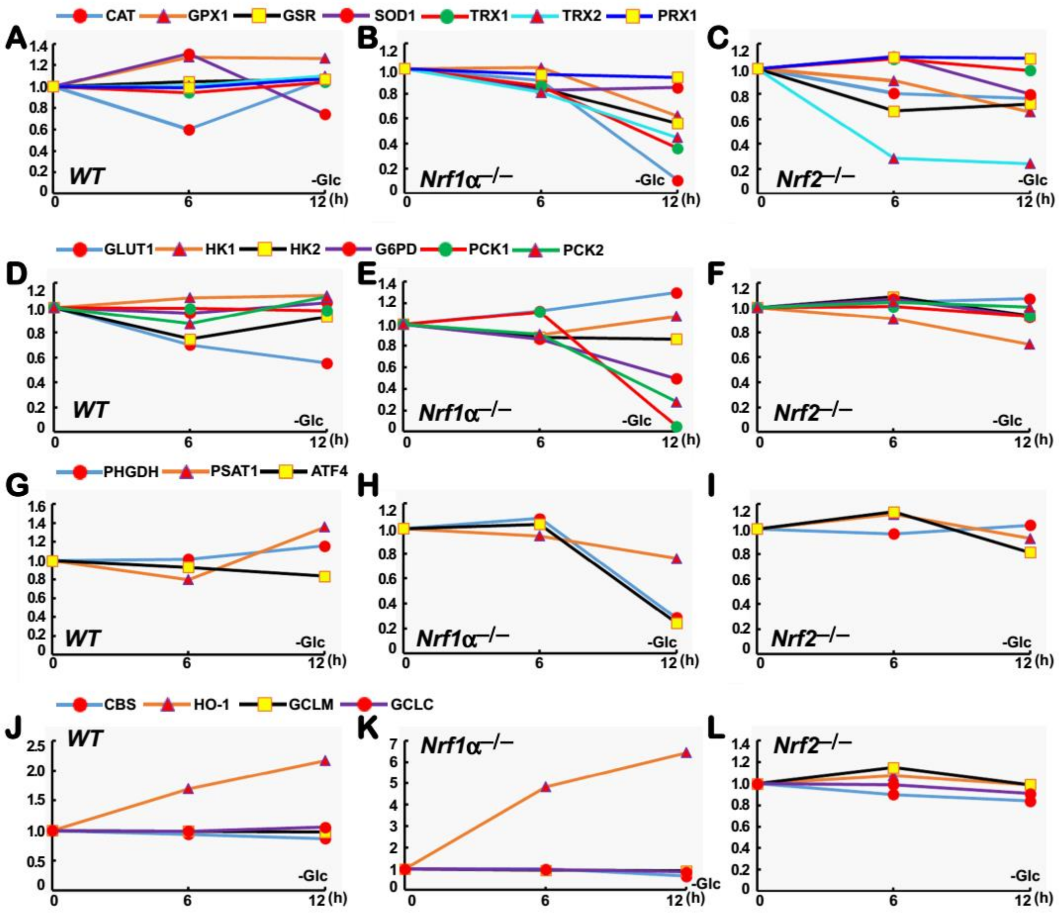

Figure S2. The quantified results of western blot data in Figure 3, 4 and 5 by Quantity One 4.5.2 software.

(A-C) According to the western blotting data in Figure 3, The relative protein abundances of the following proteins CAT, GPX1, GSR, SOD1, TRX1, TRX2 and PRX1 in *Nrf1/2*$^{+/+}$ (*A*), *Nrf1α*$^{-/-}$ (*B*) and *Nrf2*$^{-/-}$ (*C*) were quantified by Quantity One 4.5.2.

(D-F) According to the western blotting data in Figure 4, The relative protein abundances of the following proteins GLUT1, HK1, HK2, G6PD, PCK1 and PCK2 in *Nrf1/2*$^{+/+}$ (*D*), *Nrf1α*$^{-/-}$ (*E*) and *Nrf2*$^{-/-}$ (*F*) were quantified by Quantity One 4.5.2.

According to the western blotting data in Figure 5, (G-I) The relative protein abundances of the following proteins PHGDH, PSAT1 and ATF4 in *Nrf1/2*$^{+/+}$ (*G*), *Nrf1α*$^{-/-}$ (*H*) and *Nrf2*$^{-/-}$ (*I*) were quantified by Quantity One 4.5.2. (J-L)The relative protein abundances of the following proteins CBS, HO-1, GCLM and GCLC in *Nrf1/2*$^{+/+}$ (*J*), *Nrf1α*$^{-/-}$ (*K*) and *Nrf2*$^{-/-}$ (*L*) were quantified by Quantity One 4.5.2.

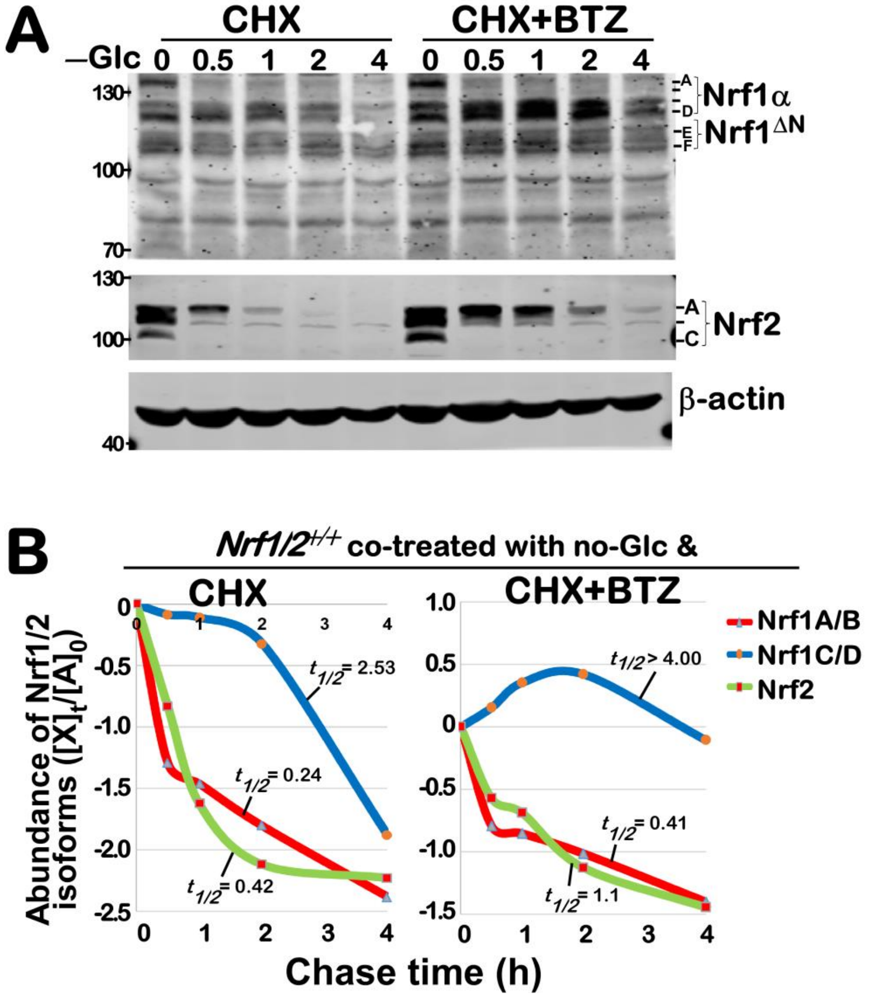

Figure S3. Time-dependent effects of glucose deprivation on Nrf1 and Nrf2 with distinct half-lives.

(A) HepG2 cells were, or were not, co-treated for 0-4 h in the glucose-free media to which cycloheximide (CHX, 50 μg/ml) alone or plus bortezomib (BTZ, 1 μmol/L resoved in DMSO) was added, before being harvested. The total lysates were resolved by SDS-PAGE gels containing 8% and 12% polyacrylamide, followed by Western blotting with antibodies against Nrf1 or Nrf2.

(B) The relative intensity of major protein bands representing Nrf1 and Nrf2 was quantified and shown graphically. The $[X]_t/[A]_0$ represent the relative amount of indicated protein at the indicated times ($[A]_t$) was normalized to $[A]_0$. The half-lives of the major Nrf1 and Nrf2 isoforms were calculated and shown herein.

Figure S4

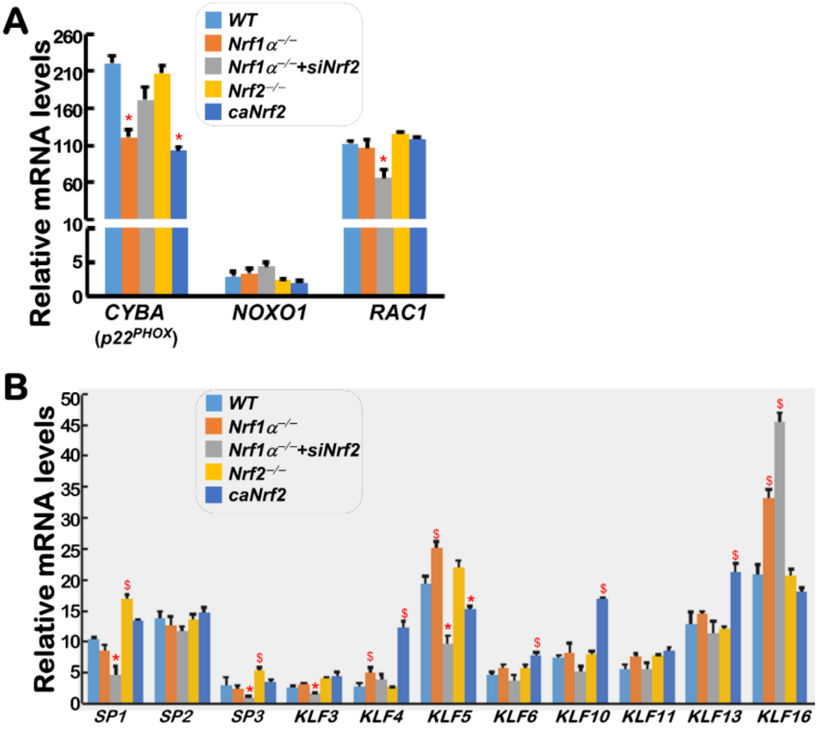

Figure S4. The mRNA expression of *CYBA*, *NOXO1* and *RAC1* in *WT*, *Nrf1α*$^{-/-}$, *Nrf1α*$^{-/-}$ + *siNrf2*, *Nrf2*$^{-/-}$ and *caNrf2* from transcriptomic sequencing (A); The mRNA expression of *SP1*, *SP2, SP3, KLF3, KLF4, KLF5, KLF6*, *KLF10*, *KLF11*, *KLF13* and *KLF16* in *WT*, *Nrf1α*$^{-/-}$, *Nrf1α*$^{-/-}$ + *siNrf2*, *Nrf2*$^{-/-}$ and *caNrf2* from transcriptomic sequencing (B). NOTE: significant increases ($, *p*<0.05) and significant decreases (**p*<0.05) were also calculated, relative to the *WT*.

Table S1. The key resources used in this work

Reagent or Resource	Source	Identifier
**Antibodies**		
GCLC	Sangon Biotechnology	D123963
CAT	BIMAKE	A5291
p^{Thr172}AMPK	BIMAKE	A5740
AMPK	BIMAKE	A5008
ATF4	BIMAKE	A5514
HO1	Abcam	ab52947
GCLM	Abcam	ab126704
GPX1	Abcam	ab108427
Nrf2	Abcam	ab62352
CBS	ABclonal	A1427
Nrf1	Zhang's	(1)
PSMB7	ABclonal	A14771
PSMB6	ABclonal	A4053
PSMB5	ABclonal	A1975
TRX2	ABclonal	A12591
TRX1	BIMAKE	A5894
PRX1	BIMAKE	A5448
SOD1	R&D Systems	AF3418
GSR	Sangon Biotechnology	D220726
PCK1	Sangon Biotechnology	D261383
PCK2	Sangon Biotechnology	D222686

G6PD	Zen-bioscience	381826
GLUT1	ABclonal	A11208
HK1	Sangon Biotechnology	D221854
HK2	ABclonal	A0994
PHGDH	ABclonal	A10461
PSAT1	ABclonal	A6707
β-actin	ZSGB-BIO	TA-09
**Chemicals**		
Trypan Blue	Solarbio	C0040
3-Methyladenine	MedChem Express	HY-19312
Ferrostatin-1	MedChem Express	HY-100579
Necrostatin-1	MedChem Express	HY-15760
Q-VD-OPH	TargetMol	T0282
Fructose	Sangon Biotechnology	A600213
Mannose	Sangon Biotechnology	A600554
DHA(Dehydroacetic acid)	Sangon Biotechnology	A602491
Catalase	Sangon Biotechnology	A001896
NAC (N-Acetyl-L-cysteine)	Sangon Biotechnology	A601127
Glutamine	Gibco	25030149
Penicillin-Streptomycin	Invitrogen	15140122
PMSF	Sangon	A100754
Protease inhibitors	Roche	3271382-1
RIPA	Beyotime	P0013C
**Oligonucleotides for small interference (si) RNA**		
Normal control FW	Tsingke	UUCUCCGAACGUGUCACGUdTdT

Normal control REV	Tsingke	ACGUGACACGUUCGGAGAAdTdT
siNrf2 FW	Tsingke	GUAAGAAGCCAGAUGUUAAdTdT
siNrf2 REV	Tsingke	UUAACAUCUGGCUUCUUACdTdT
**Oligonucleotides for qPCR**		
Nrf1 FW	Tsingke	GCTGGACACCATCCTGAATC
Nrf1 REV	Tsingke	CCTTCTGCTTCATCTGTCGC
Nrf2 FW	Tsingke	TCAGCGACGGAAAGAGTATGA
Nrf2 REV	Tsingke	CCACTGGTTTCTGACTGGATGT
CAT FW	Tsingke	TTCTGTTGAAGATGCGGCG
CAT REV	Tsingke	GATGTAAAAAGTCCAGGAGGGGT
SOD1 FW	Tsingke	ATCCTCTATCCAGAAAACACGGT
SOD1 REV	Tsingke	CAAGCCAAACGACTTCCAGC
SOD2 FW	Tsingke	AATAGCTGGGATTACAGGTGCAT
SOD2 REV	Tsingke	CCAGACGGATCACTTGAGGTCAG
GPX1 FW	Tsingke	CAGTCGGTGTATGCCTTCTCG
GPX1 REV	Tsingke	GAGGGACGCCACATTCTCG
GSR FW	Tsingke	CACGAGTGATCCCAAGCCC
GSR REV	Tsingke	CAATGTAACCTGCACCAACAATG
NOX4 FW	Tsingke	CGGCAGAGTTTACCCAGCAC
NOX4 REV	Tsingke	AAGGTCCAGAAATCCAAAGCC
TRX1 FW	Tsingke	AGCCTTTCTTTCATGATGTTGC
TRX1 REV	Tsingke	ATTGGCTCCAGAAAATTCACCC
TRX2 FW	Tsingke	CCTGACTGTAACACCCAACCC
TRX2 REV	Tsingke	CACCATCTTCTCTAACCTCGGC
PRX1 FW	Tsingke	GAAGGCATCTCGTTCAGGG

PRX1 REV	Tsingke	CCAACAGGGAGGTCATTTACA
PRX2 FW	Tsingke	GCTGTCGGACTACAAAGGGA
PRX2 REV	Tsingke	CAAGCCAGGTGGGTGAACT
CBS FW	Tsingke	CAGTTCAAACAGATCCGCCTCAC
CBS REV	Tsingke	TGTGGTACTGGATCTGCTCGTG
CTH FW	Tsingke	ACACTTCCAACATTTCGCCA
CTH REV	Tsingke	GTGCTGCCACTGCTTTTTCA
ATF4 FW	Tsingke	CCCTTCACCTTCTTACAACCTC
ATF4 REV	Tsingke	TGCCCAGCTCTAAACTAAAGGA
PHGDH FW	Tsingke	GAAATCTCTCACGGGGGTTG
PHGDH REV	Tsingke	GTTCACATCCGCCTGCTTG
PSAT1 FW	Tsingke	ACCTCAACCCAGATGCCTC
PSAT1 REV	Tsingke	TCACGGACAATCACCACGG
PSPH FW	Tsingke	TGTCCTCCTGCTGATGCTTT
PSPH REV	Tsingke	ATACCATTTGGCGTTATCCTTG
PSMB5 FW	Tsingke	GCTGTTGGCTCGGCAATGT
PSMB5 REV	Tsingke	GCCTCTCTTATCCCAGCCACAG
PSMB6 FW	Tsingke	TCAAGAAGGAGGGCAGGTGT
PSMB6 REV	Tsingke	GTAAAGTGGCAACGGCGAA
PSMB7 FW	Tsingke	CTGTGTCGGTGTATGCTCCA
PSMB7 REV	Tsingke	TGCCAGTTTTCCGGACCTTT
PC FW	Tsingke	CTGAATACTCGCCTCTTCCTGC
PC REV	Tsingke	CTTCATGGCCTGGGTGTCCTTG
PCK2 FW	Tsingke	AGTAGAGAGCAAGACGGTGAT
PCK2 REV	Tsingke	AGTAGAGAGCAAGACGGTGAT

FBP1 FW	Tsingke	CTCTATGGCATTGCTGGTTCTAC
FBP1 REV	Tsingke	GGTTCCACTATGATGGCGTGT
GLUT1 FW	Tsingke	ATTGGCTCCGGTATCGTCAAC
GLUT1 REV	Tsingke	GCTCAGATAGGACATCCAGGGTA
HK1 FW	Tsingke	CACATGGAGTCCGAGGTTTATG
HK1 REV	Tsingke	CGTGAATCCCACAGGTAACTTC
HK2 FW	Tsingke	GAGCCACCACTCACCCTACT
HK2 REV	Tsingke	CCAGGCATTCGGCAATGTG
PFKL FW	Tsingke	GGCTTCGACACCCGTGTAA
PFKL REV	Tsingke	CGTCAAACCTCTTGTCATCCA
LDHA FW	Tsingke	ATGGCAACTCTAAAGGATCAGC
LDHA REV	Tsingke	CCAACCCCAACAACTGTAATCT
GCLC FW	Tsingke	TCAATGGGAAGGAAGGTGTGTT
GCLC REV	Tsingke	TTGTAGTCAGGATGGTTTGCGA
GCLM FW	Tsingke	TGCTGTGTGATGCCACCAGA
GCLM REV	Tsingke	CGCTTGAATGTCAGGAATGCTT
NQO1 FW	Tsingke	AAGAAGAAAGGATGGGAGGTGG
NQO1 REV	Tsingke	GAACAGACTCGGCAGGATACTGA
HO-1 FW	Tsingke	CAGAGCCTGGAAGACACCCTAA
HO-1 REV	Tsingke	AAACCACCCCAACCCTGCTAT
β-actin FW	Tsingke	CATGTACGTTGCTATCCAGGC
β-actin REV	Tsingke	CTCCTTAATGTCACGCACGAT
**Oligonucleotides for construct**		
CAT-ARE#1 FW	Tsingke	cATTGCTGACTTTTTAAGAGCTGAGAAAGCATAGCTATGGAGCGCAAGGCc
CAT-ARE#1 REV	Tsingke	tcgagGCCTTGCGCTCCATAGCTATGCTTTCTCAGCTCTTAAAAAGTCAGCAATggtac

CAT-ARE#2 FW	Tsingke	cAAATCCCACCTTCTTCTTTGGCTTGCTCAAGCCTGTTCCACAGTGAACAc
CAT-ARE#2 REV	Tsingke	tcgagTGTTCACTGTGGAACAGGCTTGAGCAAGCCAAAGAAGAAGGTGGGATTTggtac
CAT-ARE#3 FW	Tsingke	cGAGACAGGGTTTTGCCATGTTGACCAGGCTGGTCTTGAACTCCTGACCTc
CAT-ARE#3 REV	Tsingke	tcgagAGGTCAGGAGTTCAAGACCAGCCTGGTCAACATGGCAAAACCCTGTCTCggtac
CAT-ARE#4 FW	Tsingke	cTGTTTTTGAGACGGAGTCTTGCTCTGTCACCCGGGCTGGAGTGCAGTGGc
CAT-ARE#4 REV	Tsingke	tcgagCCACTGCACTCCAGCCCGGGTGACAGAGCAAGACTCCGTCTCAAAAACAggtac
CAT-ARE#5 FW	Tsingke	cCCGCTGCACTCCAGCCTGGGTGACAGAGCGAGACTTCGTCTCAAAAAAAc
CAT-ARE#5 REV	Tsingke	tcgagTTTTTTTGAGACGAAGTCTCGCTCTGTCACCCAGGCTGGAGTGCAGCGGggtac
G6PD-ARE#6 FW	Tsingke	cCGGGAAGCCGGCGAGAAGTGTGAGGCCGCGGTAGGGCCGCATCCCGCTCc
G6PD-ARE#6 REV	Tsingke	tcgagGAGCGGGATGCGGCCCTACCGCGGCCTCACACTTCTCGCCGGCTTCCCGggtac
G6PD-ARE#7 FW	Tsingke	cTCCCGCTCCGGAGAGAAGTCTGAGTCCGCCAGGCTCTGCAGGCCCGCGGc
G6PD-ARE#7 REV	Tsingke	tcgagCCGCGGGCCTGCAGAGCCTGGCGGACTCAGACTTCTCTCCGGAGCGGGAggtac
G6PD-ARE#8 FW	Tsingke	cAATTTAACGACCTCGATAGAGCGCAGTCAAGTTTGGTGAACAGAATATGc
G6PD-ARE#8 REV	Tsingke	tcgagCATATTCTGTTCACCAAACTTGACTGCGCTCTATCGAGGTCGTTAAATTggtac
G6PD-ARE#9 FW	Tsingke	cACCACTGCACTCCAGCCTGGTGACAGAGCGAGATTCTGTCTCAAAAAAAc
G6PD-ARE#9 REV	Tsingke	tcgagTTTTTTTGAGACAGAATCTCGCTCTGTCACCAGGCTGGAGTGCAGTGGTggtac
G6PD-ARE#10 FW	Tsingke	cTTCTACTTGGTTATCTAGTAGCCTTCTCAAAACGAACCTCTCTAAGACCc
G6PD-ARE#10 REV	Tsingke	tcgagGGTCTTAGAGAGGTTCGTTTTGAGAAGGCTACTAGATAACCAAGTAGAAggtac
G6PD-ARE#11 FW	Tsingke	cTGCTGGCTACTCCTGCCCAGGCACTCTCACTGGGTTCCCATCCTTCATGc
G6PD-ARE#11 REV	Tsingke	tcgagCATGAAGGATGGGAACCCAGTGAGAGTGCCTGGGCAGGAGTAGCCAGCAggtac
G6PD-ARE#12 FW	Tsingke	cGACTGCCTCGGAGACTGGAGTGAGGCAGCCACAAGCCCAGGAATAAATGc
G6PD-ARE#12 REV	Tsingke	tcgagCATTTATTCCTGGGCTTGTGGCTGCCTCACTCCAGTCTCCGAGGCAGTCggtac
PHGDH-ARE#13 FW	Tsingke	cATAAAGCGGGCAGGGATTTGGCAACCTCAGAGCCGCGAGGAGGAGGCGGc
PHGDH-ARE#13 REV	Tsingke	tcgagCCGCCTCCTCCTCGCGGCTCTGAGGTTGCCAAATCCCTGCCCGCTTTATggtac
PHGDH-ARE#14 FW	Tsingke	cCATTTCTGTAAAATGGGGCATGAGAGAGCACGTCGGTTATGGGGTTTAAc

PHGDH-ARE#14 RW	Tsingke	tcgagTTAAACCCCATAACCGACGTGCTCTCTCATGCCCCATTTTACAGAAATGggtac
PHGDH-ARE#15 FW	Tsingke	cGAGCAGCCCCTGGCCCCTCTGCAGTCTCACCTCCTGGTATCCCACTGGCc
PHGDH-ARE#15 REV	Tsingke	tcgagGCCAGTGGGATACCAGGAGGTGAGACTGCAGAGGGGCCAGGGGCTGCTCggtac
PCK2-ARE#16 FW	Tsingke	cTGGATTGTGGAGAGAAAGAATGACCAGGCCGGGCGTGGTGGCTCACGCCc
PCK2-ARE#16 REV	Tsingke	tcgagGGCGTGAGCCACCACGCCCGGCCTGGTCATTCTTTCTCTCCACAATCCAggtac
PCK2-ARE#17 FW	Tsingke	cCCTTGATTCTTCCCTTAGGGTGACCGAGCAAATGACTCTTTAAGCCCGTc
PCK2-ARE#17 REV	Tsingke	tcgagACGGGCTTAAAGAGTCATTTGCTCGGTCACCCTAAGGGAAGAATCAAGGggtac
PCK2-ARE#18 FW	Tsingke	cGAAGCGGGCATTGCATTTGGTGAGACAGCTCCCTTTGGCCACAGATAGTc
PCK2-ARE#18 REV	Tsingke	tcgagACTATCTGTGGCCAAAGGGAGCTGTCTCACCAAATGCAATGCCCGCTTCggtac
ATF4-ARE#19 FW	Tsingke	cCAGAGTTTAGGAGGAGCCATGCAGACTCAGCCGGCCTTTGGGGGTAGGTc
ATF4-ARE#19 REV	Tsingke	tcgagACCTACCCCCAAAGGCCGGCTGAGTCTGCATGGCTCCTCCTAAACTCTGggtac
ATF4-ARE#20 FW	Tsingke	cAATAATAATGAAGAGTCCTCTGAGGCAGCAGGAGGATGTGATGGAGAc
ATF4-ARE#20 REV	Tsingke	tcgagTCTCCATCACATCCTCCTGCTGCCTCAGAGGACTCTTCATTATTATTggtac
ATF4-ARE#21 FW	Tsingke	cACTTTAATACTCAACCATTTGCACAGTCATCTGTCAGGAGGTACTGTCTc
ATF4-ARE#21 RW	Tsingke	tcgagAGACAGTACCTCCTGACAGATGACTGTGCAAATGGTTGAGTATTAAAGTggtac
ATF4-ARE#22 FW	Tsingke	cCCATGATACTTCCTTGGGACTGACTTGGCTGAGAACGTGTTCTGTCAGAc
ATF4-ARE#22 REV	Tsingke	tcgagTCTGACAGAACACGTTCTCAGCCAAGTCAGTCCCAAGGAAGTATCATGGggtac
ATF4-ARE#23 FW	Tsingke	cTGTCCATATTCCACATTTGCTGACTGTGCTCCCTGCACTCCACTCAAGTc
ATF4-ARE#23 REV	Tsingke	tcgagACTTGAGTGGAGTGCAGGGAGCACAGTCAGCAAATGTGGAATATGGACAggtac
ATF4-ARE#24 FW	Tsingke	cCCTGGTGGGTTTGCTGACCATGACTGGGCAAGCCGTTCTTTTTGCTGCCc
ATF4-ARE#24 REV	Tsingke	tcgagGGCAGCAAAAAGAACGGCTTGCCCAGTCATGGTCAGCAAACCCACCAGGggtac
**Recombinant DNA**		
pcDNA3.1	Invitrogen	V79020
pGL3-promoter	Promega	VQP0124
pRL-TK	Promega	VQP0126

Software and Algorithms		
Canvas 9	Cancas GFX, Inc.	https://www.canvasgfx.com/
Excel	Microsoft	https://www.microsoft.com/
FlowJo 7.6.1	FlowJo	https://www.flowjo.com/
CFX Manager 3.1	Bio-Rad	https://bio-rad-cfx-manager.com/

1. Zhang, Y., and Hayes, J. D. (2010) Identification of topological determinants in the N-terminal domain of transcription factor Nrf1 that control its orientation in the endoplasmic reticulum membrane. *The Biochemical journal* **430**, 497-510
